# Environmental Aspects of the Use of *Hedera helix* Extract in Bioremediation Process

**DOI:** 10.3390/microorganisms7020043

**Published:** 2019-02-05

**Authors:** Agata Zdarta, Wojciech Smułek, Amanda Pacholak, Ewa Kaczorek

**Affiliations:** Institute of Chemical Technology and Engineering, Poznan University of Technology, Berdychowo 4, 60-965 Poznan, Poland; wojciech.smulek@put.poznan.pl (W.S.); amanda.d.pacholak@doctorate.put.poznan.pl (A.P.); ewa.kaczorek@put.poznan.pl (E.K.)

**Keywords:** *Hedera helix* saponins, toxicity, cells properties, 4-chlorotoluene, α,α,α-trifluorotoluene, biodegradation

## Abstract

This paper analyzes the impact of saponins from English ivy leaves on the properties of environmental bacterial strains and hydrocarbon degradation ability. For this purpose, two bacterial strains, *Raoultella ornitinolytica* M03 and *Acinetobacter calcoaceticus* M1B, have been used in toluene, 4-chlorotoluene, and α,α,α-trifluorotoluene biodegradation supported by *Hedera helix* extract. Moreover, theeffects of ivy exposition on cell properties and extract toxicity were investigated. The extract was found to cause minor differences in cell surface hydrophobicity, membrane permeability, and Zeta potential, although it adhered to the cell surface. *Acinetobacter calcoaceticus* M1B was more affected by the ivy extract; thus, the cells were more metabolically active and degraded saponins at greater amounts. Although the extract influenced positively the cells’ viability in the presence of hydrocarbons, it could have been used by the bacteria as a carbon source, thus slowing down hydrocarbon degradation. These results show that the use of ivy saponins for hydrocarbon remediation is environmentally acceptable but should be carefully analyzed to assess the efficiency of the selected saponins-rich extract in combination with selected bacterial strains.

## 1. Introduction

Despite intensive works aimed at finding new, ecofriendly products of the chemical industry, some toxic compounds are irreplaceable and still in use. In addition, toluene derivatives are applied in several industrial processes, e.g., as precursors in the synthesis of functional compounds [1,2]. However, these halogenated derivatives exhibit higher toxicity and lower biodegradability than non-substituted aromatic compounds. Hence, the soil and water contamination with these compounds is especially hazardous [3].

Many applications in the removal of toxic and hazardous compounds found bioactive molecules by extending their solubilization and facilitating transport of hydrocarbons into microbial cells [4,5,6], resulting in faster and more effective elimination of pollution. The most used in environmental applications are saponins, plant-derived surfactants with amphiphilic structures that are capable of increasing both the surface tension and interfacial surface area between hydrophobic, water-insoluble liquids and water, enhancing hydrocarbon bioavailability, as well as changing the bacterial cell surface properties [7,8,9]. These properties make surfactants excellent emulsifiers and foaming and dispersing agents. Moreover, their renewable source, biodegradability, and relatively low toxicity make them a valuable alternative to chemically synthesized surfactants [10,11].

In recent years, there has been an increasing interest in the bioactive molecules from *Hedera helix* (common ivy) leaves. Early studies analyzed the antifungal [12] and antibacterial [13] activities of *H. helix* saponins. Other findings reported anti-inflammatory [14] and antileishmanial [15,16] properties of *H. helix* active compounds. Furthermore, studies of [17] proved that d-rhamnose-*β*-hederin (DR *β* -H) from Asian plant *Clematis ganpiniana* has a pro-apoptotic effect on breast cancer cells. In addition to many medical applications, hederagenin from *H. helix* was found to have applications in environmental restoration. Analysis performed by Dudášová et al. [18] and Murínová [19] with ivy leaves confirmed that addition of this material stimulates bacterial growth and activity of polychlorinated biphenyls (PCB) degradation enzymes; it also increases the biodegradation rate of PCB to 54% by *Pseudomonas stutzeri* [18] and 61% by *Ochrobactrum anthropic* [19]. Beside many promising properties of bioactive compounds in *H. helix*, it might also have an opposite effect on the environmental microcosm as ivy fruit saponins could decrease microbial community diversity because of their antibacterial properties [13,20].

Although extensive research has been carried out on *Hedera helix* medical properties, no single study exists which examines the usefulness of ivy saponins of the extract on naturally occurring bacterial strains and in toluene derivative biodegradation. Considering the prevalence of the common ivy, the presence of active compounds in easily accessible parts of the plants, as well as the significant potential of ivy-extracted saponins in environmental applications, the aim of our study was to evaluate the effect of the common ivy extract on microbial cells, its toxicity, and biodegradability. Furthermore, the influence of the addition of ivy leaves extract on toluene, 4-chlorotoluene, and α,α,α-trifluorotoluene biodegradation was examined.

## 2. Materials and Methods

### 2.1. Chemicals

Growth medium was prepared as described by Dobslaw and Engesser [21]. For saponin extraction, methanol and buthan-1-ol were used (Avantor, Poland). Hexadecane, toluene, hydrochloric acid, propan-2-ol, ONPG (2-Nitrophenyl-*β*-d-galactopyranoside), and thiazolyl blue tetrazolium bromide (MTT) were purchased form Sigma-Aldrich (Poland). All chemicals used were of analytical grade. Diesel oil was purchased from a petroleum station (PKN Orlen, Poland).

### 2.2. Microorganisms

Hydrocarbon-degrading bacteria, *Raoultella ornitinolytica* M03 and *Acinetobacter calcoaceticus* M1B, isolated from long-term, hydrocarbon-contaminated soil and obtained by enrichment in a mineral medium (pH 7.2) with diesel oil as the sole carbon source [22], were used in the experiments. The bacteria were identified genetically, and their nucleotide sequences are available in GenBank under the following accession numbers: KX.667739.1 (M03) and KU.563543.1 (M1B).

**Figure 1:**
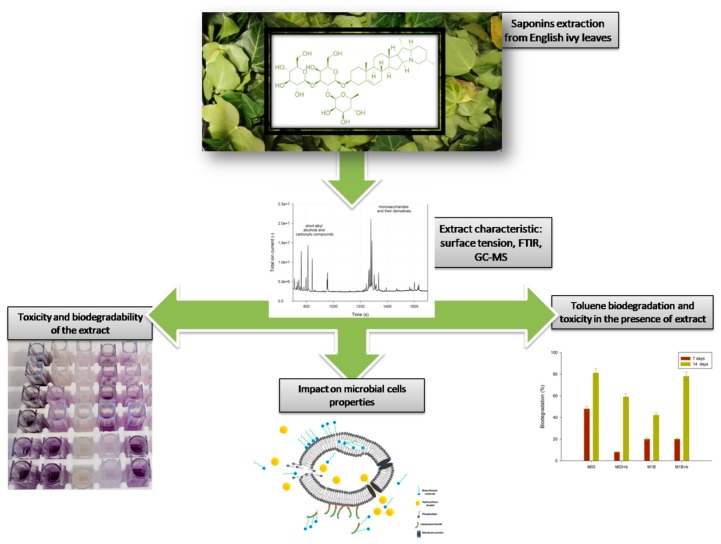
General overview of the experiments conducted with *H. helix* extract GC–MS: gas chromatography–mass spectroscopy.

Figure 1 presents a general overview of the experiments on the environmental properties of *H. helix* extract.

### 2.3. Ivy Extract Preparation and Characteristic

Saponin-rich extract was prepared as follows: approximately 60 g of cut ivy leaves of ecological cultivation origin was extracted in methanol in 250-mL, round-bottomed flasks. 10 g of cut leafs were inserted in a sample thimble and placed in a Soxhlet extractor with an Allihn reflux condenser, and 160 mL of methanol was poured into flask. The flask was heated on a laboratory electric heating mantle for 8 h. Next, the solvent was evaporated on a rotary evaporator R-210 (Buchi, Germany). For purification and saponin concentration, the extract was dissolved in MiliQ water and washed twice with hexane to remove the chlorophyll. Finally, the sample was extracted once again with butan-1-ol in a separating funnel, and the organic solvent was evaporated afterwards. Purified extract was freeze-dried for 48 h in −55 °C under a pressure of 0.37 mbar (Freeze Dryer Alpha 1-2 LD Plus, Christ, Germany). Crude extract was preserved in a desiccator. The surface tension of the water solution of the obtained extract was measured with the Du Noüy ring method on a Krüss K20 apparatus. In further experiments, water solutions of the extracts in concentration of 2 CMC (critical micelle concentration) for each extract were used. Before use, solutions were pasteurized at 65 °C for 15 min and then filtrated via 0.20 μm filter in sterile conditions. The bioactive compounds extracted from *H. helix* were analyzed by FTIR and GC–MS. For this purpose, 2 mg of lyophilized extract was mixed with 200 mg of KBr, and the disc was pressed at 10 MPa. Then, the analysis was performed using Vertex 70 (Bruker, Germany) over the wave range 4000–400 cm^−1^. The gas chromatographic analysis was conducted according to Jarzębski et al. [23]. 50 mg of extract was mixed with 0.1 mL derivatization reagent BSTFA (*N*,*O*-Bis(trimethylsilyl)trifluoroacetamide 99%, Sigma-Aldrich, Germany) and incubated (30 min, 60 °C). The qualitative analysis was conducted using the PEGASUS 4D GCxGC-TOF MS gas chromatograph (LECO Corp., St. Joseph, MI, USA) connected to a BPX5 (5% phenyl equivalent, 28 m × 0.25 mm; 0.25 μm) capillary column (SGE Int., Melbourne, Australia) with helium as a carrier gas (1.0 mLmin^−1^). After a splitless injection of 1 μL of the sample, the oven temperature was set and maintained for 2 min at 40 °C; then, it was placed on a heating ramp at 300 °C at a rate of 12 °C min^−1^. Then, the final temperature of the oven was kept for 15 min.

### 2.4. Effect of Ivy Extract on Metabolic Activity of the Cells

The impact of ivy extract on analyzed strains was investigated, as described by Walencka et al. [24]. The cell suspension of microbes grown on glucose was transferred to Eppendorf tubes (0.9 mL), an appropriate amount of surfactants was added to render the final concentration in the sample 1 g L^−1^, and the sample was then incubated for 1 h at 30 °C. An MTT reagent was added to each of the prepared samples so that its final concentration was 0.5 mg L^−1^, and they were again incubated for 24 h at 30 °C. The samples were then centrifuged for 5 min at 15,000× *g*, the supernatant was discarded, and the obtained violet precipitate was dissolved in 1 mL of propan-2-ol. The prepared samples were centrifuged again for 5 min at 5000× *g*, and the absorbance of the supernatant was measured at 560 nm. 

### 2.5. Biodegradability of the Ivy Extract

Saponin biodegradability tests were performed in sterile 250-mL glass bottles containing 45 mL of mineral salt medium, 5 mL of bacterial inoculum, and 0.05 mL of trace elements solution. The saponin concentration in the samples was 1 g L^−1^, and all samples were made in triplicate. The samples were incubated at 30 °C with shaking (120 rpm) for 27 days, and analytical samples of 1 mL were collected every 3 days in Eppendorf tubes and stored frozen until the end of the experiment. 

To evaluate the amount of saponins in the collected samples, the method proposed by Hiai et al. [25] was used. It is based on the formation of a color complex of saponins with vanillin in the presence of sulfuric acid. The intensity of the color is proportional to the amount of saponins in the sample. For this purpose, a 72% solution of sulfuric acid and 8% solution of vanillin in 99.5% ethanol were prepared. Collected samples were defrosted and centrifuged (8000× *g* for 10 min). Next, in quartz cuvettes, 1mL of sulfuric acid, 0.1 mL vanillin solution, and 0.1 mL saponin samples were mixed and incubated for 10 min in 60 °C. After that, the sample absorbance was measured at *λ* = 544 nm.

### 2.6. Impact of Ivy Extract on Microbial Cells

For cell surface properties analysis, bacteria were cultivated in 250-mLSchott-Duran^®^ flasks on a mineral salt medium (45 mL) with 5 mL of bacterial inoculum, and *H. helix* extract was added to render a final concentration of 1 g L^−1^. Cells were harvested in the late exponential phase, centrifuged at 8000× *g* for 10 min, and washed twice with a mineral salt medium. Supernatant (crude cell extract) was resuspended in the medium to fit an optical density of 1.0 at 600 nm, which was measured using Jasco V-650 UV-Vis spectrophotometer. To determine the impact of saponin-rich extract on environmental microorganisms, several parameters of the cells were examined.

Cell surface hydrophobicity was measured based on the modified microbial adhesion to hydrocarbons (MATH) method described by Kaczorek et al. [7]. Inner membrane permeability was analyzed using the ONPG test according to Zhang et al. [26]. For Zeta potential measurement, 5 mL of cell suspension was measured using a ZetaSizer Nano ZS apparatus (Malvern Instruments Ltd., Malvern, UK). Zeta potential (ξ) was determined from the electrophoretic mobility (μE) based on Henry’s equation [27]. Each of the test samples was transferred to the polystyrene, U-shaped Zeta cell. Measurements were conducted at 25 °C in triplicate, and the average for each sample was calculated. The particle size distribution of the cells suspension was determined with a Zetasizer Nano ZS apparatus (Malvern Instruments Ltd.) using the non-invasive back light scattering method (NIBS). The width parameter known as polydispersity index (PdI) was given by cumulant analysis [28].

### 2.7. Toluene Derivative Biodegradation

Bacterial inoculum was prepared by incubating each of the analyzed strains in a growth medium with 1 mL of glucose solution (20%) as a carbon source at 30 °C with shaking (120 rpm) for 48 h. After that, bacteria were used to inoculate the liquid cultures. Biodegradation was carried out in 50 mL sterile glass bottles. Each sample consisted of 18 mL of mineral salt medium with saponins (final concentration 1 g L^−1^) or without (control samples), 2 mL of bacterial inoculum, 0.02 mL of trace elements solution, and 1 mg of selected hydrocarbon solution in acetone (final concentration in samples 50 mg L^−1^). Blank samples were made without a bacterial inoculum. Each sample was made in triplicate, and samples were incubated at 30 °C for 7 or 14 days. After that, toluene residues were extracted, with 10 mL of hexane and 50 mg L^−1^ ethylbenzene as a reference standard. Bottles were closed and mixed intensively. After phase separation, approximately 1 mL of the organic-phase sample from each bottle wastransferred to chromatography vials and analyzed on Pegasus 4D GCxGC-TOFMS with BPX-5 column (28 m × 250 µm; 0.25 µm) (SGE Analytical Science, Melbourne, Australia). As a mobile phase, helium was used (1 mL min^−1^). Quantitative analysis of biodegraded compound was carried out for 13.5 min in programmed temperature growth conditions (50 °C for 3 min, then an increase of 12 °C min^−1^ to 140 °C, the final temperature was maintained by 3 min).

### 2.8. Toxicity of Hydrocarbons in Presence of the Extract

Bacterial inocula were centrifuged for 10 min at 4500× *g*. After that, supernatant was discarded, and cells were resuspended in fresh medium without or with surfactant addition (final concentration 1 g L^−1^). The cell suspension was dispensed into Eppendorf tubes (0.9 mL) and different hydrocarbons (α,α,α-trifluorotoluene, 4-chlorotoluene or toluene) were added at a concentration of 50 mg L^−1^. Samples were incubated for 2 h at 30 °C. An MTT reagent was added to each of the prepared samples so that its final concentration was 0.5 mg L^−1^, and they were incubated again for 24 h at 30 °C. The samples were then centrifuged for 5 min at 15,000× *g*, the supernatant was discarded, and the obtained violet precipitate was dissolved in 1 mL of propan-2-ol. The prepared samples were centrifuged again for 5 min at 5000× *g*, and the absorbance of the supernatant was measured at 560 nm. 

### 2.9. Statistical Analysis

All experiments were performed in triplicate (unless otherwise indicated), and the mean values were used in the calculations. Statistical analysis of the correlation of the results were performed using one-way analysis of variance (ANOVA), SigmaPlot 11.0 with *p* < 0.05.

## 3. Results and Discussion

### 3.1. Saponins Extraction and Characteristic

Saponins were extracted for 8 h in methanol, with an extraction efficiency of 20.1%. After extract purification with butan-1-ol and lyophilization, the surface tension of the aqueous solution in the air/water interface of the obtained ivy extract was determined and was equal to 39 mN m^−1^ at a concentration of 1 g L^−1^. CMC is an important parameter because below this value, accumulation of surfactant monomers at the interface can increase the contact angle between hydrocarbons and soil, modifying the wettability of the system. On other hand, surfactants over a critical micelle concentration can form micelles where hydrocarbons can be solubilized, thus increasing their partition in the solution [29]. The surface tension of the obtained extract solution is similar to the surface tension of soapnut (*Sapindus mucorossi*) extract (40 mN m^−1^) presented by Mukhopadhyay et al. [30], as well as to chemical surfactants (SDS—38 mN m^−1^; Polysorbate 80–42 mN m^−1^) reviewed by Otzen et al. [31], whereas CMC value is more comparable to sodium dodecyl sulfate (2–3 g L^−1^).

**Figure 2:**
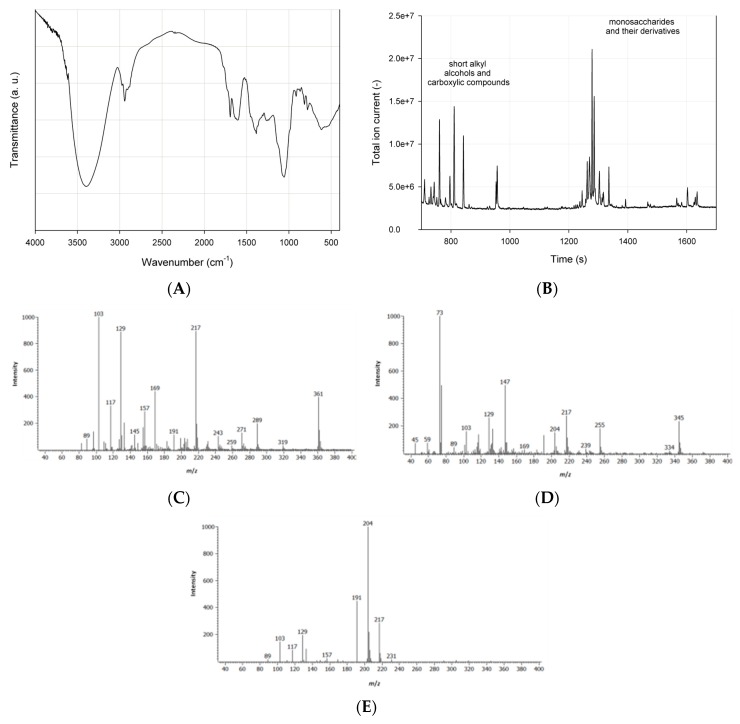
FTIR (**A**), total ion current chromatogram of saponins (**B**), and negative-mode mass spectral fragmentation patterns of the most abundant trimetylsilyl derivatives of monosaccharides of *H. helix* extract (based on GC–MS Chroma TOF-coupled MS spectra libraries): deoxyglucose (**C**), arabinose (**D**), and galactose (**E**).

The results of spectrometric analysis of the extract show adsorption bands which can be assigned to specific functional groups (Figure 2A). The adsorption bands at 3350–3420 cm^−1^ should be attributed to alcohol and acid hydroxyl groups. Alkanes are characterized by stretching and bending vibrations of C-H groups, where signals can be found in wavelength ranges 2960–2920 cm^−1^ and 1440–1430 cm^−1^. A narrow adsorption band at 1660–1650 cm^−1^ could be assigned to carbonyl group. Moreover, stretching vibrations of C-O groups can be detected at 1050 cm^−1^. The aforementioned functional groups confirm the presence of saponins in the obtained leaf extract. According to the literature, these saponin-characteristic bands were identified in infrared spectra of plants from genus *Rapanea* and *Senna* [32], *Basella* [33], and *Agave* [34]. Moreover, the presence of stretching vibrations of C=O groups suggest the structure of oleanane-type triterpenoid saponins, most likely bidesmosides [33].

The GC–MS analysis of the extract showed a high content of low-weight alkyl alcohols and carboxylic compounds as well as monosaccharides (Figure 2B). According to the GC–MS Chroma TOF MS spectra library, three main monosaccharides were identified: deoxyglucose (Figure 2C), arabinose (Figure 2D), and galactose (Figure 2E), and glucose was identified as the most dominant monosaccharide.

### 3.2. Saponin Toxicity and Biodegradability

Saponins extracted from *Quillaja saponaria* and *Chenopodium quinoa* have been registered by US EPA as active ingredients in biopesticides. Potential use of saponin-rich extract from *H. helix* in bioremediation requires insightful studies on the toxicity and biodegradability of these compounds to avoid environmental damage. For this purpose, tests of extracted saponin biodegradability and the impact on microbes’ metabolic activity were conducted. Table 1 presents results of this experiments.

As can be seen from the Table 1, the metabolic activity of the analyzed strains has grown significantly when the saponin-rich extract was added to the culture. This might be due to the presence of sugar groups in the saponin molecules that might be used by bacteria as an alternative carbon source. These results reflect those of Murakami et al. [35], who found that theaddition of saponins from *Quillaja saponaria* bark, tea seed coat, and quinoa seed did not affect Gram-negative bacteria *Vibrio fisheri*. Their further research on the acute toxicity of saponins on different organisms led to the conclusion that the biological activity of different saponin extracts may depend not only on the composition of the saponins but also on the non-saponin content of the extracts, such as tannins and polyphenol [35]. However, the presented results of ivy extract saponin biodegradability revealed that the strain *R. ornithinolytica* was able to degrade only 5% of this active compound, whereas values recorded for *A. calcoaceticus* underwent a sixfold change. Nevertheless, those values are surprisingly low considering the results presented by other scientists. Hirata et al. [36] observed a 61% degradation of *Candida bombicola* sophorolipids on the eighth day of cultivation, and Mølgaard et al. [37] found that the saponin fraction of an aqueous extract of *Phytolacca dodecandra* was completely biodegraded within 10 days in an aquatic environment under aerobic conditions. Lower than expected, saponin biodegradation rates might be the result of the presence of inhibiting substances in the extract.

### 3.3. Impact of the H. helix Extract on Microbial Cells

Cell surface hydrophobicity, membrane permeability, and electrokinetic potential are important factors affecting cell capability and hydrophobic contaminant utilization. Table 2 presents the effect of ivy extract addition on these parameters in *R. ornitinolytica* (M03) and *A. calcoaceticus* (M1B).

As shown in Table 2, cells obtained from the control sample and cultivated with saponins were hydrophilic. Moreover, surfactant addition did not change their properties to hydrophobic. Furthermore, we observed a decrease in membrane permeability after incubation with ivy extract. In the case of natural surfactants, several studies have shown that natural saponins are able to build in the cell membrane’s lipophilic part or attach to bacterial cells and thus change membrane permeability and surface charge [29]. For bacteria cultivated with saponins, a slight decrease in membrane permeability was observed for both strains, while modifications in CSH and Zeta potential were different. An addition of *H. helix* extract caused a decrease in cell surface hydrophobicity and electrokinetic potential for *R. ornithinolytica*. The extract in the same concentration generated an increase inthis parameter in the case of *A. calcoaceticus*. Those results are contrary to previous studies which have suggested that saponins increase cell membrane permeability and CSH while decreasing Zeta potential [38]. This inconsistency may be due to the complex composition of the ivy extract and the resultant impact on all of its components. For example, sapogenin content in commercially available saponin mixture from *Quillaja bark* (Sigma-Aldrich) is ≥10% according to the manufacturer. This suggests that the level of the bioactive compounds in the obtained extract is quite low; moreover, there might appear other inhibiting substances such as flavonoids, and it may significantly affect the impact of *H. helix* extract on analyzed cells.

**Figure 3:**
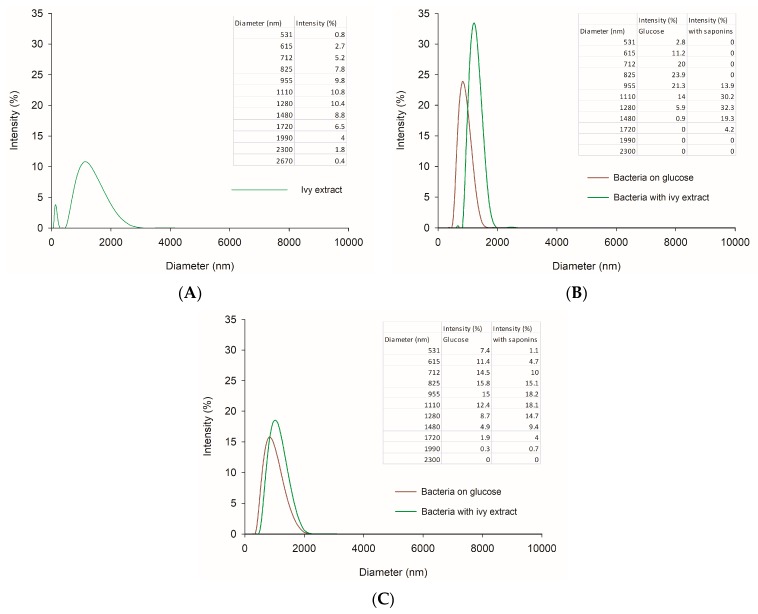
Results of particle size distribution of: ivy extract in water (**A**); and bacterial strains cultured with or without saponins: *R. ornitinolytica* M03 (**B**) and *A. calcoaceticus* M1B (**C**).

To evaluate particle aggregation and envelope by surfactant, particle size analysis was implemented. The current study found that ivy extract had a marked effect on the size of bacteria particles (Figure 3). The distribution of particles in the water solution of the extract was not homogenous as the polydispersity index (PdI) for the sample was 0.63, and the mean diameter of the particles was 1.15 μm. The measured microbial cell diameters in the control sample were mainly below 1 μm (average 0.83 μm), whereas the addition of surfactant caused an increase in analyzed particle size, with dominating particles of 1.21 μm for *R. ornithinolytica* and 0.99 for *A. calcoaceticus* (Figure 3B,C, respectively). For all analyzed bacterial samples, the polydispersity index was below 0.4, which indicates that the systems were homogenous. These results seem to be consistent with Roshtkhari and Mulligan [39], who found that the presence of rhamnolipid (0.5%), together with two microbial strains isolated from weathered oil, lead to an increase in the concentration of larger particles and particle mean diameter in the tailings samples compared to the control. Considering the relatively small shift in microbial particle diameter, it can be concluded that the observed changes in bacterial particle diameters after cultivation with surfactant suggest adhesion of bioactive compounds to the cells rather than their aggregation. The saponins present in the ivy extract might build into the cell membrane, thus modifying the size of the bacteria.

### 3.4. Biodegradation of Toluene Derivatives

**Figure 4:**
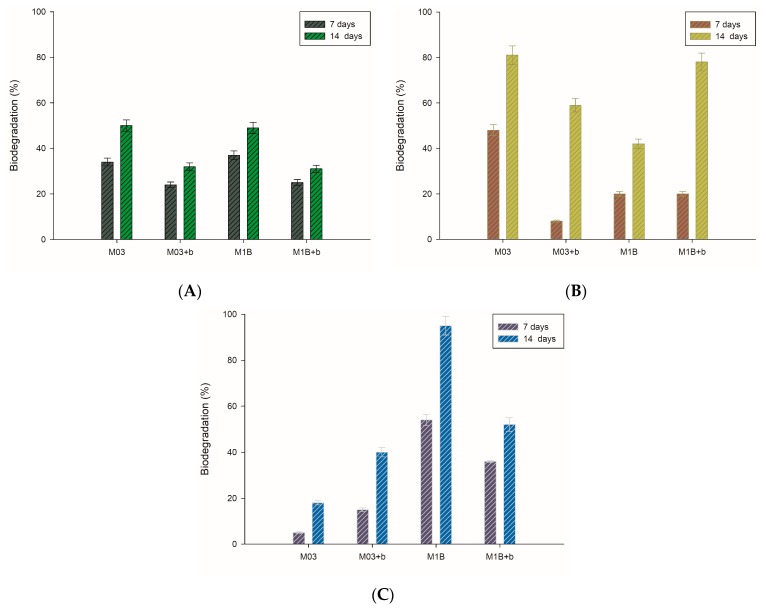
Hydrocarbons biodegradation by *R. ornithinolytica* (M03) and *A. calcoaceticus* (M1B), with (+b) or without ivy extract after 7 and 14 days; (**A**) toluene, (**B**) chlorotoluene, (**C**) α,α,α-trifluorotoluene.

Biosurfactants are promising alternatives to chemical surfactants and could be used in hydrocarbon contaminant removal. The potential of saponin-containing ivy extract to remove such compounds was evaluated during the toluene derivative biodegradation tests. Results presented in Figure 4 show the experimental data on α,α,α-trifluorotoluene, 4‑chlorotoluene, and toluene biodegradation by analyzed strains. From this data, we can see that the impact of ivy extract depends on the type of hydrocarbon and bacterial strain. A beneficial result of extract application was noticed for *A. calcoaceticus* during 4-chlorotoluene degradation (after 14 days) and α,α,α-trifluorotoluene degradation by *R. ornithinolytica*. In case of toluene degradation, no positive effect of extract addition was noticed. Woertz et al. [40] also found that sodium dodecyl sulfate (SDS) lowered toluene biodegradation rates by *Exophialalecanii-corni*, while Tween 20 increased its elimination. In accordance with the present results, previous studies of Pacholak et al. [41] have demonstrated that saponins extracted from *Sapindus mukorossi* fruits elevated the level of 4-chlorotoluene biodegradation and simultaneously negatively affected the degradation of toluene.

Microorganisms from different groups can utilize hydrocarbons as their sole source of carbon and energy. Results presented by Santos et al. [42] show that among all, bacteria from *Acinetobacter* genera were capable of degrading toluene (initial concentration 0.2% *v*/*v*) without any additives, leaving only 20% of the initial concentration after sixdays. However, the yields in this investigation were lower compared to those presented by Santos et al. [42]. As shown from the metabolic activity test, the addition of saponins promotes microbial growth and thus provides more biomass for effective hydrocarbon degradation (Table 1). On the other hand, saponins can be used by microbes as an alternative carbon source, thus negatively affecting the toluene derivatives removal. Moreover, above-critical micelle concentration saponins might cause hydrocarbon entrapment inside micelles and inhibit hydrocarbon contact with microorganisms as well as reduce the uptake of hydrocarbons due to surfactant adsorption on the microorganism’s surface [43,44].

### 3.5. Toxicity of Hydrocarbons in Presence of the Extract

The viability of the culture was determined with the MTT test for cells grown for 24 h with selected hydrocarbons as a sole carbon source and optionally additional supplementation of ivy extract. The table below (Table 3) illustrates the measured metabolic activity of *R. ornitinolytica* M03 and *A. calcoaceticus* M1B. Addition of ivy extract had a positive effect in all analyzed cases and led to an increase in the metabolic activity of the microorganisms. The most significant changes were noticed for *A. calcoaceticus* M1B, where an addition of surfactant caused a twofold increase incell activity after extract supplementation, regardless of the carbon source type. In the case of *R. ornitinolytica* M03, a positive impact of surfactant addition was noticeable, but it was not significant.

These results correspond with observations of surfactant toxicity and biodegradability presented in Table 1, where a beneficial influence of ivy extract on analyzed cells was observed. Moreover, an increase in the metabolic activity of *A. calcoaceticus* M1B might be the result of biodegradation of ivy extract components instead of hydrocarbons that is also visible as a lower level of hydrocarbon degradation in samples with saponin addition (comparing results after seven days of degradation process—Figure 3). In accordance with the present results, previous studies have demonstrated that *Aeromonas caviae* (To-4) and *Pseudomonas putida* (To-5) can grow in high concentrations of toluene in the vapour phase, and are also able to multiply in the presence of low concentrations of toluene in the liquid phase [45]. The researchers observed also that in the presence of an additional carbon source (glucose, yeast extract, and polypeptone), the growth inhibition can be overcome by toluene-induced cells. This indicates that a supplementation of the culture with a readily available carbon source might reduce the unfavourable impact of hydrocarbons, leading to high cell densities thereafter [45,46].

## 4. Conclusions

The purpose of the presented study was to determine the impact of the use of *Hedera helix* extract in the bioremediation process on environmental microorganisms. We demonstrated that the bioactive molecules in the extract have a positive effect on cell viability and might promote cell metabolic activity in the presence of hydrocarbons. Furthermore, the results suggest that the ivy extract saponins might adhere to the cells or build into cell membrane, modifying the size of bacteria. However, the addition of saponin-rich extract to *R. ornitinolytica* M03 and *A. calcoaceticus* M1B cultures in most cases does not positively affect selected hydrocarbon degradation by the used bacterial strains. Moreover, a higher bioavailability of extract compounds supports its degradation and thus might have an adverse impact on hydrocarbon biodegradation. These findings have implications when considering the application of saponin-rich extracts in the treatment of hydrocarbon-contaminated sites. Although saponin-rich ivy extract has no negative effect on analyzed microbial cells, the use of bioactive compounds in biodegradation should be preceded by careful analysis of the effect of the specified extract on the biodegradation efficiency of the tested compound.

## Figures and Tables

**Table 1:** Metabolic activity and biodegradability of saponins from ivy extract by bacterial strains: *R. ornitinolytica* (M03) and *A. calcoaceticus* (M1B).

	Metabolic Activity (%)		Saponin Biodegradability after 27 Days (%)
	Control	With Ivy Extract	
**M03**	100.0 ± 2.4	160 ± 8.0	5.0 ± 0.3
**M1B**	100.0 ± 3.1	142.0 ± 7.1	31.0 ± 1.6

**Table 2:** Effect of ivy extract addition on cell surface hydrophobicity (CSH), membrane permeability, (MP) and Zeta potential (Z) of *R. ornitinolytica* (M03) and *A. calcoaceticus* (M1B).

	CSH [%]		MP [μmol∙L^−1^min^−1^]		Z [mV]	
	Control	With Ivy Extract	Control	With Ivy Extract	Control	With Ivy Extract
**M03**	17.7 ± 0.9	7.2 ± 0.4	15.6 ± 0.8	12.1 ± 0.6	−18.6 ± 0.9	−14.7 ± 0.7
**M1B**	3.3 ± 0.2	4.7 ± 0.2	8.3 ± 0.4	6.5 ± 0.3	−11.5 ± 0.6	−16.1 ± 0.8

**Table 3:** Metabolic activity of the strains: *R. ornitinolytica* (M03) and *A. calcoaceticus* (M1B), cultivated on selected hydrocarbons with or without addition of ivy extract.

	Metabolic Activity (%)					
	α,α,α-Trifluorotoluene		4-Chlorotoluene		Toluene	
	Control	With Ivy Extract	Control	With Ivy Extract	Control	With Ivy Extract
**M03**	98.0 ± 5.2	100.4 ± 5.1	104.1 ± 5.3	101.2 ± 5.1	102.3 ± 5.0	100.0 ± 5.2
**M1B**	51.1 ± 3.3	114.0 ± 6.2	61.2 ± 3.0	113.4 ± 6.1	63.3 ± 3.2	140.1 ± 7.4
